# Driving and legal status of Spanish opioid-dependent patients

**DOI:** 10.1186/1747-597X-8-19

**Published:** 2013-06-03

**Authors:** Carlos Roncero, F Javier Álvarez, Carmen Barral, Susana Gómez-Baeza, Begoña Gonzalvo, Laia Rodríguez-Cintas, M Teresa Brugal, Carlos Jacas, Anna Romaguera, Miguel Casas

**Affiliations:** 1 Department of Psychiatry, Outpatient Drug Clinic, Vall d’Hebron University Hospital-Barcelona Public Health Agency (ASPB), 08035, Barcelona, Spain; 2 Department of Psychiatry and Legal Medicine, Universidad Autonoma de Barcelona, 08042, Barcelona, Spain; 3 Department of Psychiatry, Vall d’Hebron University Hospital, CIBERSAM, 08035, Barcelona, Spain; 4 Department of Pharmacology, Faculty of Medicine, Centre for Alcohol and Drugs Studies, University of Valladolid, Valladolid 47005, Spain; 5 Barcelona Public Health Agency (ASPB), 08023, Barcelona, Spain; 6 Institut d’Investigació Biomèdica (IIB Sant Pau), 08025, Barcelona, Spain; 7 CIBER Epidemiología y Salud Pública (CIBERESP), Madrid, Spain

**Keywords:** Legal status, Automobile driving, Traffic accidents, EuropASI, Opiate dependence

## Abstract

**Background:**

Opioid dependent patients have legal problems, driving violations and accidents more frequently than the general population. We have hypothesized that those patients currently driving may have better legal outcomes than those who do not possess a valid driving license. With this aim we have analyzed the information gathered in the PROTEUS study regarding the legal and driving statuses and assessed the possible association between them. The PROTEUS study was an observational, cross-sectional, descriptive, multicenter nationwide representative study, conducted in Spanish healthcare centers for opioid dependent patients.

**Findings:**

The driving and legal statuses of a population of opioid dependent patients ≥18 years and enrolled in Opioid Agonist Therapy treatment centers in Spain, were assessed using a short specific questionnaire and the EuropASI questionnaire to highlight distinct individual clinical needs. 621 patients were evaluable (84% men, 24.5% active workers). 321 patients (52%) drove on a regular basis. Nineteen percent of patients had some problem with the criminal justice system. There was a significant difference (p = 0.0433) in status, according to the criminal justice system, between patients who drove on a regular basis and those who did not, with a higher percentage of patients with non-pending charges among usual drivers.

**Conclusions:**

Regular drivers showed fewer legal problems than non-regular drivers, with the exception of those related to driving (driving violations and drunk driving). Driving is a good prognostic factor for the social integration of the patients and policies should be implemented to enable these patients to drive safely under medical authorization. The legal description will be useful to assess treatment efficacy.

## Background

Opioid dependent patients have frequent legal problems, which do not consist merely of drug possession and dealing, but also involve other criminal behavior, including crime against property, disorderly conduct, vagrancy, prostitution, driving violations, and more serious crimes [1-3]. It has recently been reported that among members of “narcotic anonymous” (with heroin as the main substance in use in 83.3% of patients when they approached the self-help group), 61.7% reported problems with the police and, of these, 16.7% had been imprisoned [1]. However, although addicts as a group commit a great number of crimes, they cannot be regarded as a homogeneous class [2]. Opioid dependent patients also have worse driving histories and more driving violations than the general population [4].

The EuropASI [5] is the European version of the Addiction Severity Index (ASI) [6], a commonly used instrument in daily clinical practice to get to know different aspects of the patient’s life that could have contributed to the development of the addiction. In fact, the EuropASI has been used to assess the legal situation of patients on treatment for alcohol and non-alcoholic substance addiction in several European studies. In the specific case of opioids, men were incarcerated or on parole for longer and had legal problems of a greater severity. They also had a higher EuropASI composite score in this area than women [7], as was the case for the ASI legal composite score in the United States [8]. Opioid abusers committed a higher rate of crime than cocaine addicts [9], and the legal features correlate with the EuropASI employment/support area [10].

Opioid dependent patients commit crimes and are very frequently involved in traffic accidents, causing great harm to society, as well as a large economic impact from law enforcement costs. However, related studies are still few. Knowledge of the legal and driving status can characterize the patients better and may help to identify factors that increase the risk of criminal behavior, or which may worsen the driving abilities of these patients, highlighting distinct clinical needs for individual opioid-dependent patients when presenting themselves for treatment. Furthermore, we have hypothesized that those patients currently driving may have better legal outcomes than those who do not possess a valid driving license.

We have recently carried out a multicenter nationwide representative study, the PROTEUS study [11], in opioid dependent patients enrolled in Opioid Agonist Therapy programs in Spanish care centers, describing the current therapeutic management of opiate-dependent patients undergoing such therapy programs. We have hypothesized that those patients currently driving may have better legal outcomes than those who do not possess a valid driving license. With this aim, we have analyzed the information gathered in the PROTEUS study regarding the legal and driving status and assessed the possible association between them. Specifically, we have assessed i) the frequency of current drivers among these opioid dependent patients enrolled in Opioid Agonist Therapy programs, ii) their legal status as measured through the various items of the EuropASI legal subscale, as well as through their self-rated criminal justice status, iii) the existence of differences, or not, whether the patients was a usual or non-usual driver on the EuropASI legal subscale variables, as well as on the self-rated criminal justice status.

## Methods

### Sample and recruitment

The PROTEUS study [11] was an observational, cross-sectional, descriptive, multicenter nationwide representative study, conducted in Spanish healthcare centers for opioid dependent patients. The study was approved by the Clinical Research Ethics Committee of the Vall d’Hebron University Hospital (Barcelona, Spain).

Patients of at least 18 years old diagnosed with opioid dependence, according to the Diagnostic and Statistical Manual of Mental Disorders, Fourth Edition, text revision (DSM-IV-TR) criteria [12] and enrolled in Opioid Agonist Therapy programs in Spanish care centers for patients with opioid dependence, were recruited, proportionally to the number of patients with opioid dependence registered in each Autonomous Region (17 Regions in Spain), between September 2008 and March 2009. All participants provided written informed consent before their inclusion.

### Assessment

Patient data were recorded in a single study visit, in a face-to-face interview conducted by a trained interviewer. The description of the study, as well as the variables assessed, has been presented previously [11]: The main variable referred to the current therapeutic management of patients with opioid dependence [11], such as current replacement therapy, treatment phase, dosage, time in the current replacement therapy, frequency of visits to the center, etc.

For the current study, we have analyzed the following variables:

•Usual driving of vehicles/use of machinery: No/Yes; when Yes: Work/Leisure.

•Criminal justice status: i) No pending charges ii) Imprisoned iii) Bail/probation, iv) Other: then, specify.

•The information on legal issues provided by the EuropASI legal subscale, regarding driving and legal statuses.

The Spanish translated and validated version [13] of the EuropASI questionnaire [5] was used to assess the dependence severity and related problems. The instrument has seven potential problem areas (Medical, Employment/Support Status, Alcohol, Drug, Legal, Family/Social, and Psychological), each one consisting of a number of questions on events occurring in the previous 30 days and during the patient’s lifetime. It also includes a severity rating scale determined for each area by the interviewer. The EuropASI legal area (Table 1) inquires about the number of arrests and charges for 14 kinds of offenses, has separate questions related to drunk driving charges and previous incarcerations, and can be used to assess the legal situation of the patient. Each potential problem area was scored from 0 (no problems) to 9 (extreme problems).

**Table 1:** EuropASI questionnaire (Legal area) in patients with available driving status

	**Total**	**Usual driving**	**Non-usual driving**	**p-value**^**a**^
	**N = 543**	**N = 287**	**N = 256**	
1. Was this admission prompted or suggested by the criminal justice system? YES	25 (4.7%)	11 (3.9%)	14 (5.7%)	0.4128
2. Are you on probation or parole? YES	44 (8.4%)	18 (6.5%)	26 (10.7%)	0.1136
3. How many times in your life have you been charged for possession and dealing of drugs?	0.76 ±1.85	0.61 ±1.68	0.93 ±2.02	0.0020
4. How many times in your life have you been charged for crime against property?	1.87 ±6.92	1.14 ±4.29	2.70 ±8.93	< 0.0001
5. How many times in your life have you been charged for crimes of violence?	0.29 ±1.59	0.19 ±0.82	0.41 ±2.16	0.0286
6. How many times in your life have you been charged for other crimes?	0.40 ±2.41	0.22 ±1.07	0.61 ±3.35	0.3478
7. How many of these charges resulted in convictions?	1.12 ±3.39	0.77 ±2.65	1.51 ±4.04	< 0.0001
8. How many times in your life have you been charged with disorderly conduct, vagrancy or public intoxication?	0.34 ±1.40	0.17 ±1.02	0.54 ±1.72	< 0.0001
9. How many times in your life have you been charged with prostitution?	0.04 ±0.34	0.02 ±0.23	0.05 ±0.44	0.3667
10. How many times in your life have you been charged with driving while intoxicated?	0.16 ±0.84	0.24 ±1.09	0.07 ±0.36	0.0014
11. How many times in your life have you been charged with major driving violations?	0.38 ±1.71	0.57 ±2.25	0.15 ±0.64	0.0003
12. How many months were you incarcerated in your life?	13.20 ±33.66	9.73 ±29.67	17.09 ±37.31	< 0.0001
13. If yes in q.12, how long was your last incarceration? (mo.)	8.71 ±19.55	6.64 ±17.68	10.90 ±21.18	< 0.0001
15. Are you presently awaiting charges, trials or sentences? YES	76 (15.1%)	32 (11.9%)	44 (18.8%)	0.0339
17. How many days in the past 30 were you detained or incarcerated?	0.30 ±2.45	0.35 ±2.92	0.24 ±1.78	0.2914
18. How many days in the past 30 have you engaged in illegal activities for profit?	0.37 ±2.34	0.15 ±1.91	0.64 ±2.74	0.0002
19. How serious do you feel your present legal problems are? (Patient rating scale)	0.63 ±1.20	0.48 ±1.02	0.79 ±1.35	0.0043
20. How important to you now is counseling or referral for these legal problems? (Patient rating scale)	0.72 ±1.31	0.63 ±1.24	0.83 ±1.37	0.0431
21. How would you rate the patient’s need for legal services or counseling? (Interviewer severity rating scale)	1.28 ±1.97	1.05 ±1.77	1.55 ±2.14	0.0106
22. Is the above information significantly distorted by the patient’s misrepresentation? YES	29 (5.5%)	7 (2.5%)	22 (9.0%)	0.0017
23. Is the above information significantly distorted by the patient’s inability to understand? YES	10 (1.9%)	4 (1.4%)	6 (2.5%)	0.5256

Data expressed as mean ± SD or n (%), for continuous or categorical variables, respectively.

Only patients with available data were considered in the analysis; i.e. patients with missing data were not included in the total, and thus, N may vary in each variable.

^a^. Fisher exact test or non-parametric Mann–Whitney test for categorical and continuous variables, respectively.

### Measures and analyses

Descriptive statistics were obtained for all analyzed variables on i) EuropASI legal subscale, ii) driving and iii) criminal justice status. In a subsequent analysis, the EuropASI legal subscale variables (as well as the criminal justice status) were analyzed, depending on whether the patients were a usual or non-usual driver. Chi square and T tests were used to compare the characteristics of the sample when required. All analyses were performed using the number of valid cases (N) for each variable. Statistical analyses were performed using the SAS program (Statistical Analysis System), version 9.1.3. For all comparisons, a statistical significance level of 0.05 was considered.

### Overall patient characteristics

Six hundred and twenty-four patients were enrolled in this study and 621 were evaluable, since 3 did not comply with study inclusion criteria [11]. This patient population consisted of 84% men, 83% with social support (patients who live with their family of origin or own family or friends), mostly (94%) in methadone maintenance programs at a mean dose of 61.52 mg/day, and a high prevalence of psychiatric (67%) and infectious diseases (59%; mainly HCV, HIV and both). A total of 35% of patients had a family history of opioid abuse, usually from siblings (76%); eighty-two percent of patients were abusing drugs (27% heroin and 4.5% other opioids) at the time of the study; most patients (73%) had been previously included in a prior Opioid Agonist Therapy program for an average of 11.6 years; and 24.5% of patients were active workers [11].

## Findings

### Driving and legal status

Of the 624 enrolled patients, 617 were evaluable for driving status, 321 (52.0%) drove vehicles/used machinery on a regular basis and although the reason for driving was not specified for 85 patients (26.5%), the reason was work for 40 (12.4%) and leisure for 196 (61.1%).

Six hundred and nine patients were evaluable for their criminal justice system status. Eighty-one percent had no pending charges (n = 493), 6.7% were on bail/probation (n = 41), 2 patients were imprisoned (0.3%), and 12.0% (n = 73) were in another legal status, mainly pending trial (8.2%, n = 50).

### The EuropASI legal situation

The legal situation, according to the EuropASI questionnaire, of opioid-dependent patients enrolled in an Opioid Agonist Therapy program in Spain is described in Table 1. A total of 543 patients responded to all questions of the EuropASI legal subscale, and only these cases were considered in the analysis. Admission to the program was prompted or suggested by the criminal justice in only 4.7% of cases as the result of a conviction; while, in the rest of the cases, the seeking of treatment was voluntary. The most frequent major crime for which patients were charged was crime against property, with a mean of 1.87 times in their lifetime. Crimes of violence had a mean of 0.29 times in a lifetime. Patients were charged with drunk driving a mean of 0.16 times in their life and with major driving violations the mean was of 0.38 times. Patients had been incarcerated a mean of 13.2 months in their lifetime, and in the previous month, they had been detained or incarcerated 0.30 days and had been engaged in illegal activities for profit 0.37 days. Fifteen percent of patients were awaiting charges, trial or sentence. Only 7.4% of patients might have given significantly distorted information due to misrepresentation (29 patients) or inability to understand (10 patients).

### Driving and the EuropASI legal situation

Usual drivers committed significantly fewer crimes (Table 1, questions 3, 4, 5, 7, 8, lifetime period, and question 18, previous 30 days period) than non-drivers, and spent significantly less time incarcerated (questions 12 and 13, life time period). At the time when the survey was carried out, a smaller percentage was awaiting charges (question 15), and their legal problems were less severe (questions 19, 20 and 21) among those opiate dependent patients who drove than those who did not.

As expected, in the life time period, usual drivers were charged with drunk driving and with major driving violations significantly more times than non drivers (questions 10 and 11, Table 1).

### Associations between driving and legal status

There was a significant difference (p = 0.0433), according to the criminal justice system, between the status of patients who drove on a regular basis and those who did not, with a higher percentage of patients with non-pending charges among usual drivers (Table 2). However, among drivers, the status did not change whether patients drove for work or for leisure (p = 0.3579, data not shown).

**Table 2:** Status regarding the criminal justice system of patients with available driving-related and EuropASI data

	**Total**	**Usual driving**	**Non-usual driving**	**p-value**^**a**^
	**N = 543**	**N = 287**	**N = 256**	
Non-pending charges	427 (80.6%)	239 (84.5%)	188 (76.1%)	0.0433
Imprisoned	1 (0.2%)	1 (0.4%)	0	
Bail/Probation	35 (6.6%)	14 (4.9%)	21 (8.5%)	
Other status	67 (12.6%)	29 (10.2%)	38 (15.4%)	

^a^. Fisher exact test.

Values expressed as n (%) with respect to total patients with available data (i.e., not including patients with missing data, 13 total, 4 usual drivers, 9, non-usual drivers; thus, N may vary for each variable).

## Discussion

The PROTEUS study, a nationwide representative study of opiate dependent patients on treatment in Spain, shows that 1) legal problems are frequent, although eight out of ten patients included in the study had no pending charges at the moment of the interview, patients had been charged, during the lifetime period, 0.76 times with possession/dealing drugs, 1.87 with crime against property, 0.29 with crimes of violence, and 0.40 with other crimes. 2) Over half the patients included in the study were currently drivers. 3) Regular drivers showed fewer legal problems, spent less time incarcerated and their legal problems were less severe, than non-regular drivers, with the exception of those related to driving (driving violations and drunk driving).

Nineteen percent of the patients had some problems with the criminal justice system. This proportion of patients with legal problems is much smaller than in other European countries, such as the Netherlands, where, among the heroin users applying voluntarily for a methadone program, 42% of patients had legal problems for which they required help [according to the revised Addiction Severity Index (ASI-R)] [14].

Over half the patients (52%) in centers for opioid dependent patients on Opioid Agonist Therapy drove vehicles/used machinery on a regular basis, 2 out of 3 mainly for leisure. There is an increased awareness of the role of drugs in driving [15]. Although most actions are focused on the general driving population, this must be extended to the dependent patients. First, because they are ill (dependence and frequent psychiatric co-morbidity) and, in accordance with worldwide regulations [16], fitness to drive evaluation of such patients is needed. Furthermore, medication prescribed for the dependence disorder is impairing to fitness to drive [17]. In the current study, 94% of the patients were in treatment with methadone at average doses of over 60 mgs/day, which impair driving. Making the situation worse, some opiate dependent patients also use illegal drugs. The illness, opioid dependence, prescribed medication and cognitive impairment/fitness to drive are a controversial issue. Overall, patients perform better on treatment than without treatment, and although all medicines for opioid dependence are rated as level II/III [17] impairing medicines, not all medicines available on the market are similar (methadone, buprenorphine, etc.). Finally, opioid dependent patients have worse driving histories (driving violations and accidents), higher than the general population [4,18,19].

Health professionals treating these opioid dependent patients should be aware of these issues and should adequately inform their patients: Additional measures should be taken to make patients aware of the implications of driving while on treatment. Also, an alternative treatment with fewer effects on the driving ability of the patient might be considered, especially in those patients who drive for work, for whom non-driving is out of the question. Patients on buprenorphine have shown better decision-making ability [20,21] and better performance in attention, including reaction time, verbal memory [22] and psychomotor tests [23] than patients on methadone; all of which point to better cognitive abilities for driving in the first group of patients.

What follows is the information to be provided and recorded in the clinical records regarding medicines, driving and fitness to drive from the European Union DRUID project [24]: “WP 7 Partners have discussed that in situations where physicians will advise a patient to start driving again after a period in which the advice was given not to drive while using the medicine, specific procedures are recommended to structure the consultation and to manage the risk of litigation in case an accident could occur.

Recommendation 8: It is recommended that the following actions are taken during the consultation:

1. Advise not to combine (psychotropic) medication without the advice of a physician or pharmacist and to avoid the combination with alcohol.

2. Check whether the patient is willing and able to follow the treatment plan and explain the patient’s liability in case the patient is non-compliant to the treatment plan.

3. Advise the patient to be aware of possible side-effects and to refrain from driving in case these side-effects occur.

4. Advise the patient to report on these side-effects during a follow up visit.

And furthermore documentation of the following items in the patient’s medical record:

1. Tests performed and/or information gathered in assessing fitness to drive.

2. Assessment of patient’s decision-making competence based on advice given.

3. Patient’s understanding of impairing properties of the medication.

4. Specific actions to achieve fitness to drive (changes in medication or instructions for use).

5. Follow up visit for evaluation of interventions (advice given, self assessment of patient)” [24].

Opioid Agonist Therapy programs have resulted in a marked reduction in drug use, with a significant positive impact, among other variables, on the patient’s criminal behavior [1]. Therefore, these programs are not only able to manage medico-health issues but also specific sociopolitical problems, such as illicit drug use and crime [25]. Several factors, such as prior criminal activity and employment status, as well as treatment compliance and length, seem to be associated with post-treatment outcome in terms of illicit drug use and criminality [1]; thus the importance of assessing the patient’s status regarding the predicting variables before treatment, and of ensuring compliance. Knowing these factors opens up the possibility of adjusting treatment length depending on the individual patient’s risk and applying additional therapy/measures if necessary. The success of treatment, measured in crime reduction, also has important economic implications. Treatment reduces the costs of drug-related crime, criminal justice costs and theft by 4 to 7 dollars per dollar invested, and when health care savings are added in, total savings can exceed costs by a ratio of 12 to 1 [26].

The limitations of the study have already been mentioned in a previous report [11]. However, regarding the current study, it should be mentioned that information on driving was limited, lacking information on other key issues like exposure (km driven).

In conclusion, more than half the opioid dependent patients in Spanish centers drove on a regular basis, despite being on methadone Opioid Agonist Therapy, and usual drivers showed more frequent drunk driving and major driving violations than non-usual drivers. However, driving was associated with fewer legal problems, since it is probably a normalization factor for the patient, and in some cases, necessary for his/her work. Additional measures should be taken to ensure safe driving in these patients. Driving is a good prognostic factor for the social integration of the patients and policies should be implemented to enable these patients to drive safely under medical authorization. Healthcare professionals should be aware of this problem and become more involved, providing patients with the information and necessary prescription [24]. The legal status was assessed and can be used to measure the effectiveness of the Opioid Agonist Therapy programs applied and to compare this population with other groups of opioid dependent patients.

## Competing interest

Dr. Roncero has received honoraria for speaking for: Janssen-Cilag, Bristol-Mayers Squibb, Pfizer, Reckitt Benckiser, Lundbeck, Servier and Adamed Spain; and he has received fees for participating as a member of the Janssen-Cilag and Shire board.

Dr. Álvarez has no conflict of interest.

Dr. Barral has collaborated as a speaker for Bristol-Myers Squibb, and has received funds from Adamed for funding a Mentalization Based Therapy Training.

Ms. Susana Gómez-Baeza has no conflict of interest.

Dr. Begoña Gonzalvo has no conflict of interest.

Ms. Laia Rodríguez-Cintas has no conflict of interest.

Dr. M. Teresa Brugal has no conflict of interest.

Dr. Carlos Jacas has no conflict of interest.

Dr. Anna Romaguera has no conflict of interest.

Dr. Casas has received funds from Janssen-Cilag, Pfizer, Adamed, Reckitt Benckiser and AstraZeneca laboratories and he has received a fee for participating as a member of the Janssen-Cilag board.

## Authors’ contributions

All authors contributed to the interpretation of the data, and to drafting and revising the present manuscript. All authors read and approved the final manuscript.

## References

[B1] Nurco DN Ball JC Shaffer JW Hanlon TE The criminality of narcotic addicts J Nerv Ment Dis 1985 173 94 102 10.1097/00005053-198502000-00006 3881559

[B2] Raftopoulos A Flora K Substance use related behavior of the members of narcotics anonymous and alcoholics anonymous in greece J Psychoactive Drugs 2011 43 238 244 10.1080/02791072.2011.605705 22111407

[B3] EMCDDA Drugs and crime — a complex relationship 2007 Lisbon: EMCDDA Drugs in focus Issue: 16. http://www.emcdda.europa.eu/html.cfm/index36331EN.html

[B4] Vasic G Mihajlovic G Jovanovic-Mihajlovic N Rafajlovic M Barisic J Djukic Dejanovic S Jankovic S Radonjic K Differentiation between opiate addicts in relation to judicial problems Srp Arh Celok Lek 2011 139 Suppl 1 52 56 22352204

[B5] EUROPASI Working Group European version of the addiction severity index (EUROPASI) 1994 http://www.emcdda.europa.eu/html.cfm/index3647EN.html

[B6] McLellan AT Kushner H Metzger D Peters R Smith I Grissom G Pettinati H Argeriou M The fifth edition of the addiction severity index J Subst Abuse Treat 1992 9 199 213 10.1016/0740-5472(92)90062-S 1334156

[B7] Holscher F Reissner V Di Furia L Room R Schifano F Stohler R Yotsidi V Scherbaum N Differences between men and women in the course of opiate dependence: is there a telescoping effect? Eur Arch Psychiatry Clin Neurosci 2010 260 235 241 10.1007/s00406-009-0053-x 19838765

[B8] Wu LT Ling W Burchett B Blazer DG Shostak J Woody GE Gender and racial/ethnic differences in addiction severity, HIV risk, and quality of life among adults in opioid detoxification: results from the national drug abuse treatment clinical trials network Subst Abuse Rehabil 2010 1 13 22 21709734 10.2147/SAR.S15151 PMC3122483

[B9] García Rodríguez O Secades Villa R Fernández Hermida J Carballo Crespo J Errasti Pérez J Al-Halabi Díaz S EuropASI comparison of cocaine and heroin addicts Adicciones 2005 17 33 42

[B10] Iraurgi Castillo I Sanz Vazquez M Martinez-Pampliega A Familyfunctioning and addiction severity in persons that request treatment Adicciones 2004 16 185 195

[B11] Roncero C Fuste G Barral C Rodríguez-Cintas L Martínez-Luna N Eiroa-Orosa FJ Casas M Therapeutic management and comorbidities in opiate-dependent patients undergoing a replacement therapy programme in Spain: the PROTEUS study Heroin Addict Relat Clin Probl 2011 13 5 16

[B12] American Psychiatric Association Diagnostic and statistical manual of mental disorders Text Revision 2000 4 Washington, DC: American Psychiatric Association

[B13] Bobes J Bascarán M Bobes T Carballo J Díaz E Flórez G García-Portilla M Sáiz P Assessment of addiction severity: application to treatment management and monitoring 2007 Madrid: Ministerio de Sanidad y Política Social

[B14] Meulenbeek PA Addiction problems and methadone treatment J Subst Abuse Treat 2000 19 171 174 10.1016/S0740-5472(00)00116-1 10963928

[B15] Schulze H Schumacher M Urmeew R Auerbach K Alvarez FJ Bernhoft IM de Gier JJ Hagenzieker M Houwing S Knoche A Pilgerstorfer M Zlender B Driving under the influence of drugs, alcohol and medicines in europe — findings from the DRUID project 2012 Lisbon: EMCDDA http://www.emcdda.europa.eu/publications/thematic-papers/druid

[B16] Council Directive 91/439/EEC of 29 July 1991 on driving licences http://eur-lex.europa.eu/LexUriServ/LexUriServ.do?uri=CELEX:31991L0439:EN:HTML

[B17] Ravera S Monteiro SP de Gier JJ van der Linden T Gómez-Talegón T Alvarez FJ DRUID project WP4 partners. A european approach to categorizing medicines for fitness to drive: outcomes of the DRUID project Br J Clin Pharmacol 2012 74 920 931 10.1111/j.1365-2125.2012.04279.x 22452358 PMC3522805

[B18] Alvarez FJ Gomez-Talegon T Marcos A Accident rates for drug-dependent patients in treatment for substance dependence: a pilot trial Traffic Inj Prev 2010 11 460 465 10.1080/15389588.2010.492844 20872300

[B19] Perez K Santamarina-Rubio E Rodriguez-Martos A Brugal MT Ricart I Suelves JM de la Torre R Pujadas M Ariza C Diez E Nebot M Ramos P Martinez Beneyto V Plasencia A Substance use among non-fatally injured patients attended at emergency departments in Spain Drug Alcohol Depend 2009 105 194 201 10.1016/j.drugalcdep.2009.06.023 19674852

[B20] Pirastu R Fais R Messina M Bini V Spiga S Falconieri D Diana M Impaired decision-making in opiate-dependent subjects: effect of pharmacological therapies Drug Alcohol Depend 2006 83 163 168 10.1016/j.drugalcdep.2005.11.008 16343811

[B21] Soyka M Limmer C Lehnert R Koller G Martin G Kufner H Kagerer S Haberthur A A comparison of cognitive function in patients under maintenance treatment with heroin, methadone, or buprenorphine and healthy controls: an open pilot study Am J Drug Alcohol Abuse 2011 37 497 508 10.3109/00952990.2011.600381 21851203

[B22] Rapeli P Fabritius C Alho H Salaspuro M Wahlbeck K Kalska H Methadone vs. buprenorphine/naloxone during early opioid substitution treatment: a naturalistic comparison of cognitive performance relative to healthy controls BMC Clin Pharmacol 2007 7 5 17565668 10.1186/1472-6904-7-5 PMC1914339

[B23] Soyka M Hock B Kagerer S Lehnert R Limmer C Kuefner H Less impairment on one portion of a driving-relevant psychomotor battery in buprenorphine maintained than in methadone-maintained patients: results of a randomized clinical trial J Clin Psychopharmacol 2005 25 490 493 10.1097/01.jcp.0000178417.60426.60 16160628

[B24] de Gier H Heissing M Alvarez J Tant M Recommendations for improving medical guidelines for assessing fitness to drive in patients who use psychotropic medicines DRUID project deliverable 7.2.1., Revision 2.0 2009 http://www.druid-project.eu/cln_031/nn_107548/Druid/EN/deliverales-list/downloads/Deliverable__7__2__1,templateId=raw,property=publicationFile.pdf/Deliverable_7_2_1.pdf

[B25] Bennett C Methadone maintenance treatment: disciplining the ‘addict’ Heal Hist 2011 13 130 157 10.5401/healthhist.13.2.0130 22329263

[B26] Centers for disease control and prevention (CDC) Substance abuse treatment for injection drug users: a strategy with many benefits. National center for HIV, STD and TB prevention. Prevention among injection drug users (IDU). Fact sheet series 2002 http://www.cdc.gov/idu/facts/Treatment.htm

